# Pulmonary Hypertension in Left Ventricular Valvular Diseases: A Comprehensive Review on Pathophysiology and Prognostic Value

**DOI:** 10.3390/life13091793

**Published:** 2023-08-22

**Authors:** Nikolaos Velidakis, Elina Khattab, Evangelia Gkougkoudi, Nikolaos P. E. Kadoglou

**Affiliations:** Medical School, University of Cyprus, Palaios Dromos Lefkosias Lemesou No. 215/62029 Aglantzia, P.O. Box 20537 1678, Nicosia 2024, Cyprus

**Keywords:** pulmonary hypertension, valvular disease, mitral stenosis, mitral regurgitation, aortic stenosis, aortic regurgitation

## Abstract

Left ventricular (LV) valvular diseases, make up one of the most common etiologies for pulmonary hypertension (PH), and it is not well understood how and at which degree it affects prognosis. The aim of the present study was a comprehensive review of the pathophysiologic mechanism of PH in patients with LV valvular diseases and the prognostic value of baseline and post-intervention PH in patients undergoing interventional treatment. The pathophysiology of PH in patients with LV valvular diseases involves gradual elevation of left ventricular filling pressure and left atrial pressure, which are passively transmitted to the pulmonary circulation and raise pulmonary artery systolic pressure (PASP). A long-lasting exposure to elevated PASP progressively leads to initially functional and thereafter irreversible structural changes in the pulmonary vasculature, leading up to high pulmonary vascular resistance. Surgical treatment of severe LV valvular diseases is highly effective in patients without resting PH or those with exercise-induced PH (EIPH) before intervention. In the case of pre-operative PH, successful interventional therapy decreases PASP, but the post-operative cardiac and all-cause mortality remain higher compared to patients without pre-operative PH. Hence, it is of paramount importance to detect patients with severe LV valvulopathies before the development of PH, since they will get greater benefits from early intervention.

## 1. Introduction

Pulmonary hypertension (PH) is defined as an elevated pressure in the pulmonary arterial network. According to the 6th World Symposium on Pulmonary Hypertension, a mean pulmonary arterial pressure (mPAP) above 20 mmHg sets the diagnosis of PH, which replaced the previous definition of a cut-off value at 25 mmHg [1,2]. The additive measurements of high pulmonary capillary wedge pressure (PCWP) [≥15 mmHg) and pulmonary vascular resistance (PVR) [≥3 Wood units] may assist to distinguish the origin of pulmonary vascular dysfunction by classifying PH into pre-capillary, isolated post-capillary, and combined pre- and post-capillary [2]. The diastolic pressure gradient (DPG) is the difference between the diastolic pulmonary artery pressure and the PCWP. It is another hemodynamic marker, since DPG ≥ 7 mmHg has been proposed to distinguish isolated post-capillary and combined pre- and post-capillary PH. It is also associated with poor prognosis in patients with PH [3,4]. In clinical terms, there is a long-standing classification of PH into five groups, according to the aetiology, pathophysiological mechanisms, clinical presentation, hemodynamic findings, and lastly, therapeutic management. The five groups are: (1) pulmonary arterial hypertension (PAH), (2) PH associated with left heart disease, (3) PH associated with lung diseases and/or hypoxia, (4) PH associated with pulmonary artery obstructions, and (5) PH with unclear and/or multifactorial mechanisms [4]. Among the aforementioned categories the PH associated with left heart disease is the most common cause of PH worldwide [5].

The most common symptoms of patients with PH are exertional dyspnea and fatigue, which gradually progress to symptoms and signs of right ventricular failure, namely chest pain, peripheral edema, pleural effusion, ascites, elevated jugular venous pressure, syncope, and others when right ventricular (RV) failure is developed [6]. The most frequently used modalities for PH diagnosis include transthoracic echocardiography (TTE), cardiopulmonary exercise testing (CPET), and right heart catheterization (RHC).

TTE makes up the first-line modality to screen patients with clinical suspicion of PH [4,6]. A thorough TTE examination provides an estimation of the pulmonary artery systolic pressure (PASP), calculated by the maximum tricuspid regurgitation velocity (TRVmax), which has shown a high correlation with PASP calculation during RHC. Hence, TTE remains a feasible, highly available, reproducible, valid, and cheap method with high specificity and sensitivity for PH diagnosis [7,8]. According to the 2022 guidelines from the European Society of Cardiology and European Respiratory Society, a TRVmax > 2.8 m/s is suggestive of PH presence [4]. From the Bernoulli equation we can calculate PASP. A value greater than 40 mmHg is considered to be the lower threshold of PH, which is further quantified into mild (40 mmHg ≤ PASP < 50 mmHg), moderate (50 mmHg ≤ PASP < 60 mmHg), and severe PH (PASP ≥ 60 mmHg) by most experts [9]. However, PASP measurement may be inaccurate in patients with established lung disease, such as chronic obstructive pulmonary disease (COPD) or pulmonary interstitial disease, which may blur the degree and origin of PH in a significant number of patients, especially in those with co-existing heart failure (HF) [10]. Moreover, PASP calculation as an index of the pressure gradient between the right atrium (RA) and RV is determined by the absolute pressures in both cavities. Thereby, PASP may be underestimated in the case of RV failure, where right intraventricular pressure is amplified.

Exercise-induced pulmonary hypertension (EIPH) is defined as an exaggerated increase in mPAP above 30 mmHg and PVR above 3 Wood units during exercise, while at rest the mPAP is below 25 mmHg [11]. It was initially proposed as an early index of PH development, unmasking subtle pulmonary dysfunction. In the due course of time, studies, notably of weak evidence, reported significant confounders and since 2008 the usage of EIPH was not further recommended. Nowadays, a growing body of evidence has remarkably supported the re-introduction of EIPH in the diagnostic algorithm of PH. EIPH is considered by many scientists as a precursor stage of PH, which means that earlier initiation of treatment may be related to better prognosis [12]. Exercise stress echocardiography (ESE) has been proposed for the assessment of PASP during exercise testing (treadmill or ergocycle). A maximum elevation of PASP ≥ 60 mmHg at the end of the exercise test or an abrupt elevation of PASP at a low workload of the exercise test have been proposed as indices of EIPH [13]. Moreover, diastolic dysfunction grade III at rest or the development of diastolic dysfunction of any degree under gradually increasing physical stress contribute to PASP elevation [14]. The assessment of diastolic dysfunction is based on the calculation of the ratio of early diastolic trans-mitral flow velocity to early diastolic mitral annular velocity (E/e’). ESE has become a valid modality for EIPH diagnosis, since it has a good correlation with invasive measurements. CPET is another useful diagnostic tool for EIPH, especially for the investigation of exercise intolerance in patients without resting PH. However, its availability is limited to a few specialized centers worldwide, staffed by scientists with adequate training and high levels of expertise [15]. Most importantly, CPET provides cardiorespiratory measurements along resting and exercise-induced alterations in pulmonary pressures [16]. Novel data imply that CPET may be useful for early diagnosis or prognosis estimation of PH [17,18]. 

By default, resting RHC remains the gold standard method for a firm diagnosis of PH, where calculated mPAP exceeds the cut-off value of 20 mmHg, as previously mentioned. Although it is an invasive technique, there are less confounders comparing to TTE. RHC during exercise has been proposed as the most valid method for EIPH diagnosis, since it provides direct measurements of mPAP during the exercise test. A small number of studies have demonstrated a good relationship between invasive (RHC) and non-invasive modalities (ESE, CPET) for EIPH detection. The latter tests are preferably used as the first-line for the investigation of unexplained dyspnea, when there is a mismatch between resting PASP calculation and clinical suspicion. A possible strategy in a patient with a clinical suspicion of EIPH is to start the investigation with a non-invasive modality, likely ESE, and if the suspicion persists, proceeding to RHC during exercise is recommended. A rapid saline infusion may trigger an abnormal increase in PCWP ≥ 18 mmHg, suggestive of heart failure with preserved ejection fraction (HFpEF). That fluid challenge may unmask left ventricular (LV) diastolic dysfunction in patients with mismatch between HF symptoms and PCWP measurements (≤15 mmHg), when RHC during exercise is not available [19]. In addition to this, the uncovering of occult postcapillary PH provides a prognostic estimation value in the short and long term [20].

**Pathophysiology:** PH as a consequence of left heart diseases is the most common form of PH and it is associated with poor prognosis and increased morbidity and mortality [21]. The pathophysiology of PH in LV valvular diseases involves a multitude of processes, characterized by elevated LV filling pressure (LVFP) and left atrial pressure (LAP), which is passively transmitted backwards to pulmonary circulation and raises the PASP [22]. A long-lasting exposure to elevated pressures progressively leads to: (a) functional and structural irreversible changes in the pulmonary vasculature, (b) increases in pulmonary vascular resistance (PVR), and (c) eventually, RV failure and death [23]. 

**Figure 1:**
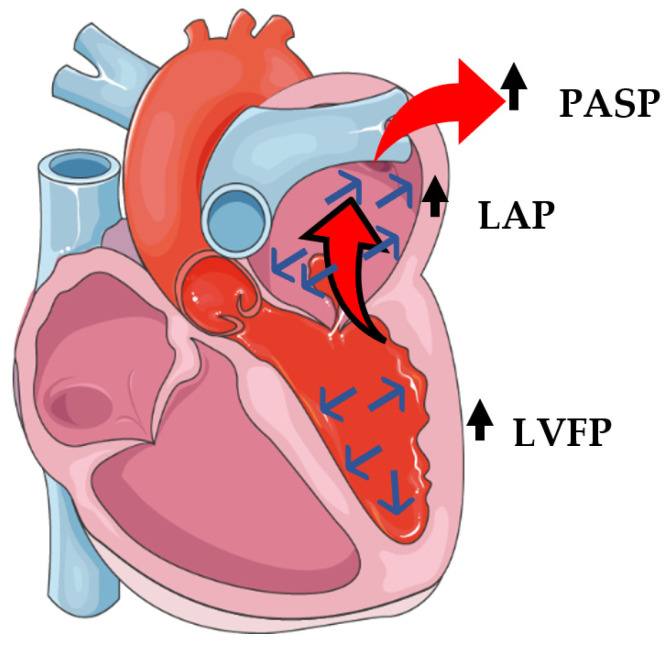
Hemodynamic changes in patients with severe mitral regurgitation: increased left ventricular pressure, backward blood flow from the left ventricle to the left atrium leading to elevation of the left atrium pressure. The latter is passively transmitted to pulmonary veins and pulmonary circulation leading to pulmonary hypertension. Red arrow: passive backward pressure transmission from pulmonary veins to pulmonary circulation 🡪 PASP elevation, Red-black arrow: backward blood flow, Blue arrows: increased left ventricular filling pressure, PASP: pulmonary artery systolic pressure, LAP: left atrial pressure, LVFP: LV filling pressure.

The development of PH in chronic severe mitral regurgitation (MR) is associated with volume overload in the LV and left atrium (LA) [24], which initially leads to enlarged LA without significant elevation in mean LAP [21]. In the advanced decompensated phase of chronic MR, there is a progressive decline in LV contractility, a maladaptive increase in LV dimensions with increased systolic wall stress, and an elevation of LVFP [21]. On the top of those aforementioned disorders, there is a systolic backward blood flow into the LA through the mitral valve, which results in rapid elevation of LAP and PCWP, and eventually leads to the development and deterioration of PH [25] (Figure 1).

In patients with mitral stenosis (MS), the LA emptying is limited causing a progressive increase in LAP, before a significant enlargement of LA has been established [26]. LV diastolic dysfunction and reduced LA compliance accompanied with increased LAP leads to a corresponsive pressure elevation into the pulmonary veins, and eventually increased PASP [22]. The severity and the underlying pathogenetic mechanisms may determine the reversibility of PH after treatment. For example, in case of PH due to volume overload, as in patients with concomitant significant MS and MR, a longer time would be required for PASP restoration after intervention. On the other hand, a rapid reduction in PASP is observed in MS without remarkable LA dilatation after interventional relief of stenosis [27].

In aortic stenosis (AS) and aortic regurgitation (AR) the increased LAP is caused indirectly due to LV systolic and diastolic dysfunction [23]. The LV dysfunction in aortic valve diseases derives mainly from LV pressure overload in AS and LV pressure and volume overload in AR [22]. In severe AS, pressure overload leads to maladaptive LV concentric thickening/hypertrophy, which is associated with LV diastolic dysfunction. Gradually LVFP increases, LA dilates, and LAP eventually elevates [23]. A common finding among AS patients is the association of PASP with the severity of the AS (measured by aortic valve area (AVA)) or the reduced LV ejection fraction (LVEF) [23]. A long-standing presence of severe AS results in LV and LA morphological and functional changes. This explains the delayed, over-time reduction in PASP after interventional treatment rather than an immediate decline observed in patients with severe AS, but without LV and/or LA severe dilatation [23]. In AR, the combined LV volume and pressure overload results in LV dilatation, eccentric hypertrophy, and LV systolic and diastolic dysfunction. The latter causes LA enlargement, functional MR, increased LAP, and eventually, PH [23] (Figure 1).

EIPH in LV valvular diseases reflects exercise-triggered hemodynamic changes and its presence can be a premature indicator of the severity of LV valvulopathy [12]. EIPH seems to derive from increased LVFP and LAP upon exertion [12]. A backward transmission of elevated LVFP and LAP to the pulmonary circulation explains the increase in PASP during exercise [28]. Besides this, the backward blood flow from the LA to pulmonary circulation in patients with MR is augmented during exercise due to the exaggerated movement of mitral valve annulus. Thereby, at least moderate MR contributes to EIPH leading to pulmonary congestion [12]. 

Nevertheless, there are several, still unknown, factors that may additionally play a role in the pathogenesis of PH, including genetic predisposition. This is supported by the fact that many patients with chronic, severe either MR, AS, or AR do not develop PH [21,23]. The initial phase of elevated PASP in LV valvular disease is an exaggerated response to abrupt LAP elevation, leading to isolated post-capillary PH (ipc-PH) [22]. This stage is still reversible and it is characterized by normal PVR and a normal transpulmonary pressure gradient (TPG: mPAP—PCWP) [21]. Long-standing elevated PASP or repeated episodes of EIPH lead to irreversible changes and the remodeling of pulmonary vessels, including collagen deposition and an imbalance in endothelial production of vasoactive mediators [21]. Pulmonary vasoconstriction and PVR progression result in the gradual worsening of combined pre- and post-capillary PH (cpc-PH) [22]. Most importantly, the development of functional and morphological changes in the pulmonary vasculature may explain the disproportional elevation of PASP at an early stage of ESE in patients with MR, despite the absence of PH at rest. The elevated PASP and PVR represent an increased RV afterload, leading to hypertrophy, dilatation, and dysfunction of the RV myocardium, and the development of TR and RV failure [29].

## 2. Left Ventricle Valvular Diseases and Pulmonary Hypertension

### 2.1. Aortic Stenosis and Pulmonary Hypertension

**Prevalence of resting PH and EIPH in AS:** The prevalence of PH in patients with AS varies significantly among studies due to the variance in population characteristics, diagnostic modalities, and cut-off values. Only a few studies have used the recently updated cut-off value of 20 mmHg for mPAP, regarding the diagnosis of PH [1]. Other studies were conducted using the cut-off value of mPAP ≥ 25 mmHg. Two recent, small, observational studies implicated a PH prevalence of 32% and 54%, respectively, among patients with severe AS [30,31]. A recent systematic review and additional older studies have estimated the range of PH prevalence between 48 and 78% [32,33,34]. However, their design differed (randomised controlled trials, prospective or retrospective studies) and they enrolled heterogeneous cohorts at baseline. Alternatively, many studies have used the non-invasive PASP measurement derived from TTE, for the diagnosis of PH. They have shown an even greater variance in the range of estimated prevalence (between 6% and 71%), which is partly explained by the use of different cut-off values of PASP for PH diagnosis [30,35].

Similarly, the prevalence of EIPH also differed among a few available studies and ranged between 55% and 90%, depending on the diagnostic method [28,33,36,37]. In particular, the prevalence of EIPH tended to be even higher using RHC during exercise, in comparison to ESE modality.

**Prognostic value of resting PH and EIPH in AS:** Many studies have investigated the prognostic power of PH among patients with AS. A meta-analysis of 22 studies enrolling candidates for transcatheter aortic valve implantation (TAVI) demonstrated an increased risk for post-intervention cardiac and all-cause mortality (HR: 1.8, 95% CI: 1.3–2.3; HR: 1.56, 95% CI: 1.1–2, respectively) in patients with pre-intervention PH compared to those patients without PH [35]. It is important to mention that pre-intervention PASP > 60 mmHg was associated with worse outcomes in the aforementioned meta-analysis. More recent studies using the proposed cut-off limit mPAP ≥ 20 mmHg have consistently indicated an increased 1-year mortality rate after TAVI, compared to counterparts without pre-intervention PH [29]. However, the authors stressed the fact that this association was mainly driven by patients with a mPAP > 25 mmHg rather than those with mPAP between 20–25 mmHg. According to a recently published systematic review, TAVI yielded a significant decline in PASP measurements, which surprisingly was not associated with significantly reduced mortality at 30 days and a one-year follow-up [30,38]. Other studies have reported the association of persistent post-TAVI PH with worse all-cause and cardiovascular mortality at 30 days, one year, and 5.9 years maximum follow-up time [39]. On the other hand, the reduction of PASP after TAVI correlated only with reduced incidence of atrial fibrillation (AF), severe MR, and LVEF < 30% [40].

In the cases of patients with severe AS undergoing aortic valve replacement (AVR), the presence of PH at baseline was associated with reduced intra-hospital and long-term survival [41,42]. The 1-year and 5-year mortality rates were linearly related to the severity of PH [39,43,44]. For example, patients with severe AS and concomitant PH, as defined by PASP ≥ 60 mmHg, had a 2.4-times-greater risk of death at 5 years in comparison to counterparts without PH, despite the alleviation of PH after AVR (HR: 2.4, 95% CI: 1.3–4.2). On the other hand, the persistence of PH after AVR was associated with AS severity leading to a worse long-term prognosis [40]. The aforementioned findings indicate that PH presence at baseline is an index of poor prognosis in patients with AS undergoing interventional therapy. The significant amelioration of PH after TAVI or AVR improves prognosis, but to a lesser extent than those without baseline PH at all. Thereby, a timely diagnosis of even low-grade PH in patients with severe AS may lead to an earlier intervention with greater benefit for this group of patients. On the other hand, no data are available about the prognostic power of EIPH in severe AS, perhaps due to the known exercise limitations in those patients.

### 2.2. Aortic Regurgitation and Pulmonary Hypertension

#### 2.2.1. Prevalence of Rest PH and EIPH in AR

Limited data are available regarding the prevalence of PH in patients with AR. A retrospective study of 506 patients with severe AR estimated the prevalence of severe PH (PASP > 60 mmHg) to be 16%, using TTE [45]. An older study of 150 patients with severe AR undergoing cardiac catherization identified 64% of patients with PH, as defined by PASP ≥ 30 mmHg [46]. In the same study, the prevalence of severe PH (PASP ≥ 60 mmHg) was 24%. Despite a thorough search, we could not retrieve more and newer studies regarding this topic.

#### 2.2.2. Prognostic Value of Resting PH and EIPH in Patients with AR

A prospective study in 332 patients with severe AR undergoing AVR identified PH as an independent predictor of 2-year and 4-year prognosis [47]. In particular, the presence of PH prior to AVR was associated with an 8.2- and 7.2-times-higher risk for the 2-year and 4-year mortality rates, respectively, compared to those without PH. Despite the adverse impact of PH presence in patients with severe AR, the performance of AVR still improved the 1-year and 5-year survival rates compared to patients following only conservative treatment (1-year survival: 90% vs. 58%; 5-year survival: 62% vs. 22%, respectively) [43]. Such favourable effects were not examined in relation to pulmonary pressure changes. In another, older study with a relatively small number of participants, the presence of PH did not affect the early post-operative outcomes following AVR, while the pulmonary pressures returned to near-normal levels in the majority of patients. [44]

Overall, in patients with severe AR undergoing AVR, the co-existence of severe PH seems to adversely affect the mortality rate. A prompt AVR may be safe and effective, but we need larger, randomized controlled trials to draw firm conclusions.

### 2.3. Mitral Stenosis and Pulmonary Hypertension

#### 2.3.1. Prevalence of Resting PH and EIPH in MS

Only a limited number of studies has examined the prevalence of PH in patients with MS. Among 317 patients with significant MS undergoing percutaneous balloon mitral commissurotomy (PBMC), the prevalence of PH was estimated up to 73% [48]. Interestingly, an elevated TPG was five times more common in women compared to men. Less common, but at a considerable level (38%), the prevalence of at least moderately severe PH was found among 559 patients with severe MS undergoing mitral balloon valvuloplasty (MBV) [49]. In this study PASP with a cut-off of 50 mmHg was used for moderately severe PH definition. Another retrospective study reported a prevalence of 25% of severe PH (defined as PASP ≥ 60 mmHg) among patients with severe MS [50].

#### 2.3.2. Prognostic Value of Resting PH and EIPH in Patients with MS

According to the current European Society of Cardiology (ESC) guidelines for valvular diseases at resting PASP > 50 mmHg in asymptomatic patients with significant MS (MVA ≤ 1.5 cm^2^) is considered a Class IIa recommendation for PMBC, when it is not contra-indicated [51]. Due to the limited number of small studies, it is still unclear whether PH affects the prognosis of patients with significant MS. Based on retrospective data, the short-term efficacy of MBV in patients with severe MS is influenced neither by the presence of baseline PH [52], nor by the significant lowering of PASP after the procedure (from 79 to 36.7 mmHg, *p* < 0.001). However, restenosis-free survival was lower in the PH group at the 10- and 15-year follow-up. A recent study examined both the short- and long-term prognosis of patients with MS and PH who underwent a surgical replacement [53]. Moderate or severe PH was associated with a worse 30-day prognosis in comparison to normal PASP or mild PH (12% vs. 5%, respectively). Additionally, the 12-year survival was considerably higher in patients without PH or mild PH than those with moderate or severe PH (79% vs. 51%). An old study examined how the co-existence of severe or mild PH affects the prognosis in patients with severe MS undergoing percutaneous MBV [54]. The results showed similar rates of severe complications after the procedure between the two groups. In agreement with this, a retrospective study showed a similar success rate and clinical course of percutaneous MBV between patients with high and lower values of PASP [48]. Regarding the TPG before PBMC and its influence on prognosis, patients with MS and normal TPG showed a marginally better short-term outcome in comparison to those with elevated TPG (75% vs. 62%, respectively) [46]. Notably, both groups had similar improvement in the mitral valve area and NYHA functional class at 36 months.

From the methodological point of view, most data have been derived from old studies, using the previous definition for PH, while the incidence of MS has remarkably shrunk in developed countries. Regarding the effective prevention and treatment of rheumatic fever, we did not expect large studies about the course of MS and PH in the near future. It would be wise to get more data from countries with a high incidence of MS in order to clarify the prognostic role of PH in those patients. 

A large cohort study was conducted in Australia investigating the predictive role of the persistence of PH after MV replacement (MVR) in patients with either MR or MS [55]. Postoperative PH was observed in 64.1% of patients (7042/10,994), as has been defined by PASP calculated to be above 40 mmHg. Postoperative PH was associated with greater all-cause mortality in comparison to patients without PH (41.1% vs. 26.3%). However, those data should be considered with caution, since they were based on a registry of a mixed population (MS or MR) undergoing MVR and the percentage of patients with PH before MVR was not reported. Whether PH development is reversible in patients with severe MS adapting to the high LAP remains to be proved. Currently, we do not have robust evidence that PH presence has a significant impact on mortality and morbidity rates in patients with MS undergoing interventional treatment. 

### 2.4. Mitral Regurgitation and Pulmonary Hypertension

**Prevalence of resting PH and EIPH in primary MR:** The presence of PH in patients with MR is usually associated with the: severity of valvular disease, worsening of symptoms, diastolic dysfunction, and higher pre- and post-operative morbidity and mortality [20,56]. The prevalence of PH in patients with severe MR ranges from 20–30%, and it increases up to 64% in severe symptomatic patients with New York Heart Association Class (NYHA) III-IV [57], and further up to 78% in patients requiring mitral valve surgery [58]. Although resting PH has been recorded in a minority of patients with severe primary asymptomatic MR [55,59], the exercise test can unravel the presence of EIPH in a larger proportion of patients (up to 58%) [56]. Thus, EIPH is more frequent than resting PH in patients with primary MR and its presence may be related to potential adverse outcomes [55]. 

#### 2.4.1. Prognostic Value of Resting PH and EIPH in Primary MR

According to the current European Society of Cardiology (ESC) guidelines for valvular diseases, a resting PASP > 50 mmHg in patients with primary severe MR is considered a Class IIa recommendation for surgical intervention [49], despite the increased peri-operative risk [57,60]. Accumulated evidence suggests that, in patients with severe MR, the new onset of PH further increases the risk of HF and death [61,62]. Especially among candidates for MV surgery, previous studies showed that the presence of pre-operative PH or the persistence of PH post-operatively was associated with reduced LVEF, increased HF-related hospitalizations, and mortality [59,63,64,65,66,67,68]. A meta-analysis of 11 studies recruiting 2011 patients examined the impact of PH on the post-operative survival in patients with severe MR undergoing trans-catheter mitral valve repair (TMVr) and demonstrated the significant relationship of baseline PH with increased mortality post-operatively [69]. In another large retrospective study of 4071 patients undergoing TMVr for severe MR, most of them with primary MR, the severity of pre-intervention PH was associated with increased hospitalizations due to HF, adverse clinical outcomes, and all-cause mortality [70]. Most importantly, the presence of EIPH in patients before undergoing surgical intervention for MR has been related to adverse survival outcome, LV dysfunction, limited reverse LV remodelling, and insufficient symptoms improvement post-operatively [71]. This raises concerns about resting PASP threshold (>50 mmHg) in the current guidelines. According to them, intervention is recommended in MR patients late in the disease course. Presumably, patients with “asymptomatic” severe MR may obtain a greater benefit from early intervention in the early stages of resting PH.

There is a consensus that PASP ≥ 60 mmHg during exercise is an important cut-off value with a negative prognostic significance in patients with severe MR [69,72,73]. In the past American College of Cardiology/American Heart Association guidelines, exercise-induced PASP ≥ 55 mmHg was considered a Class IIa indication for intervention in asymptomatic patients with MR [58]. However, this indication was removed in 2014 due to a lack of evidence [58]. Žvirblytė R et al., suggested that the elevation of PASP ≥ 60 mmHg during ESE can unmask symptoms and subclinical LV dysfunction in otherwise-called “asymptomatic” patients [74,75]. It seems that EIPH is common in patients with primary moderate to severe MR and preserved LVEF, and its presence is associated with resting LV diastolic dysfunction [76], as well as an increased risk of adverse cardiac events following MVR [70]. In another study of 123 patients with moderate to severe MR undergoing conservative treatment alone, the highest TRVmax and/or PASP levels during ESE were related to poor prognosis [74]. Those authors also showed a linear association between the values of PASP at low and peak workloads. Thus, the assessment of PASP only at low workload, which is easily obtained, may be adequate and clinically applicable [77]. Overall EIPH is considered a predictor of low survival and symptoms occurrence [70,71,72]. Importantly, it seems that an early rise of PASP during mild exercise is a significant predictor of symptoms’ development and morbidity in the short-term follow-up [40]. Current guidelines comment that ESE is as an additional test in patients with asymptomatic severe MR, which is used to unmask symptoms and detect EIPH [51,77,78]. The presence of increased PASP during exercise may be useful for risk stratification and therapeutic management of patients with MR [55,77].

**Prevalence of resting PH and EIPH in secondary MR**: Severe secondary MR is a common finding among patients with HF and reduced ejection fraction (HFrEF) [55]. The diagnosis of PH is established in approximately 40% of patients with LV dysfunction and secondary MR [55], while its prevalence further increases up to 70% in patients admitted with acute HF [79]. In case of HFpEF, PH largely co-exists and portends a poor prognosis [80,81]. However, several conditions may contribute to the high prevalence of PH in HFpEF patients and, not necessarily to the presence of secondary MR. Based on limited data, EIPH is observed in up to 40% of patients with secondary MR [82], and its presence usually predicts the occurrence of symptoms of HF decompensation and death [55,77]. It seems that in secondary MR, the dynamic increase of MR severity during exercise is a key determinant of EIPH presence, limiting further the functional capacity of HF patients [82].

#### 2.4.2. Prognostic Value of Resting PH and EIPH in Secondary MR

The Cardiovascular Outcomes Assessment of the MitralClip Percutaneous Therapy for Heart Failure Patients with Functional Mitral Regurgitation—COAPT trial showed reduced rates of hospitalization and improved survival in patients with HF and moderate to severe secondary MR undergoing TMVr (MitraClip), plus additionally receiving guideline-directed medical therapy (GDMT) [83]. Patients receiving conservative treatment alone served as controls. An analysis from the COAPT trial demonstrated that increased PASP at rest was associated with a progressively worse prognosis and a higher 2-year risk of hospitalization or death despite the intervention [84]. Also, PASP was reduced within the first 30 days following TMVr and the reduction was independently associated with reduced 30-day or 2-year rates of HF-related hospitalization or death [85]. The finding that significant resting PH (PASP > 50 mmHg) is related to adverse clinical outcomes at the 2-year follow up in patients with HF and secondary MR, agrees with the results from previous single-center registries [69,81,86]. Matsumoto et al., demonstrated that in patients with secondary MR, TMVr with MitraClip improved the severity of the valvulopathy and symptoms [87]. However, the presence of pre-operative PH was related to increased all-cause mortality after the procedure [88]. Another prospective study examining the hemodynamic impact of TMVr with MitraClip in patients with HF and secondary MR in association with different types of PH, showed that patients with ipc-PH had the most favorable post-operative hemodynamic outcomes, including improved cardiac output and RV function [84]. All related results for each valvulopathy have been summarized in Table 1.

## 3. Discussion

An elevation of mPAP > 20 mmHg measured in the resting RHC sets the diagnosis of PH [4]. EIPH is considered by many scientists as a precursor stage of PH and it is defined as a rise in mPAP > 30 mmHg and PVR > 3 WU during exercise [11,12]. The most frequently used modalities for PH diagnosis include TTE, RHC, and in a few specialized centers, CPET [4,15]. ESE is recommended as an additive modality for EIPH detection, since it has a good correlation with invasive measurements and may be useful, especially in the presence of a mismatch between a patient’s symptoms and the severity of the valvulopathy [49].

Increased LVFP and LAP, which are passively transmitted backwards to pulmonary circulation and eventually raise PASP, are essential parts of the pathophysiology of PH in LV valvular diseases [20]. In the long term, this eventually leads to irreversible changes in the pulmonary vasculature and an increase in PVR [21]. Τhere are some differences in the pathophysiologic mechanisms depending on the valve disease. It seems that MS is very often associated with PH, as opposed to AR in which it is not so often observed.

The prevalence of PH and EIPH in LV valvular diseases mostly results from a small number of studies with a small number of participants, and varies significantly among them, thus there is a lack of homogeneous data. For instance, the prevalence of PH in patients with severe primary MR is 20–30%, and it increases up to 64% in severe symptomatic patients (NYHA III-IV) [55], and further up to 78% in patients presenting for mitral valve surgery [56]. EIPH is observed more frequently than resting PH in patients with primary MR (up to 58%) [56]. High resting mPAP or EIPH in LV valvulopathies is significantly associated with the severity of the valvular disease, increased pre- and post-operative morbidity, and mortality [19,20,54]. It is therefore very important to ensure early detection of elevation in mPAP for risk stratification. Intensive management of these patients is required, before the development of irreversible damage in pulmonary vasculature occurs, which has a worse prognosis compared to counterparts without PH at baseline [20,54].

Most importantly, the presence of pre-operative PH is associated with increased post-operative cardiac and all-cause mortality, despite the amelioration of PH after intervention [36,59,61,62,63,64,65,66]. Furthermore, the presence of persistent post-operative PH and the development of RV dysfunction with severe PH are negative prognostic factors in patients undergoing valvular surgery [51]. It is therefore of paramount importance to detect patients with significant LV valvular diseases, without PH or with elevated PASP during exercise, because they will obtain prognostic benefits from early intervention.

## Figures and Tables

**Table 1:** Summary of the relationship of each valvular disease with hemodynamic changes and prognosis in patients with concomitant PH at baseline.

Comparison of All Parameters with Patients without Concomitant PH at Baseline										
Valvular Disease	Pre-Intervention						Post-Intervention			
	LA		LV		Lungs		PASP Reduction	Survival		
	LAP	Dimensions	Dimensions	LVFP	PVR	PASP		PH Absence	Transient PH/PASP > 60 mmHg	Persistence PH
MR	↑↑	↑↑	↑↑	↑↑	↑↑	↑↑	√	↓	↓↓	↓↓↓
MS	↑↑↑	↑↑	-	↓	↑↑	↑↑↑	√	-	-	↓
AS	↑↑	↑	-	↑↑	↑	↑	√	↓	↓	↓↓
AR	-	-	↑↑	↑↑	↑↑	↑	√	-	-	↓

MS: Mitral Stenosis, MR: Mitral Regurgitation, AS: Aortic Stenosis, AR: Aortic Regurgitation, LAP: Left Atrium Pressure, PVR: Pulmonary Vascular Resistance, PASP: Pulmonary Arterial Systolic Pressure, LA: Left Atrium, LV: Left Ventricle, PH: Pulmonary Hypertension, LVFP: Left Ventricular Filing Pressure. Number of arrows depict amount of change, ↑: increase, ↓decrease, -: no significant change, √: PASP reduction achieved.
